# 745. Title: Epidemiology of Hospital-onset Bacteremia (HOB) caused by Methicillin-resistant *Staphylococcus aureus* (MRSA) in a Multi-hospital Health System

**DOI:** 10.1093/ofid/ofad500.806

**Published:** 2023-11-27

**Authors:** Harjot K Singh, Barbara Ross, Margaret Quinn, David P Calfee, Matthew Simon, Heidi M Torres, Karen P Acker, Harold Horowitz, Tina Z Wang, Nuwan Gunawardhana, Robin H Goldberg, Sorana Segal-Maurer, Nishant Prasad, Candace L Johnson, David Kuang, Adam L Gouveia, E Yoko Furuya, Karen Westervelt, Lisa Saiman

**Affiliations:** Weill Cornell Medicine, new york city, New York; NewYork-Presbyterian Hospital, New York, NY; New York Presbyterian Hsopital, Malverene, New York; Weill Cornell Medicine, new york city, New York; Weill Cornell Medicine, new york city, New York; Weill Cornell Medicine, new york city, New York; Weill Cornell Medicine/New-York Presbyterian, New York, New York; New York Presbyterian-Brooklyn Methodist Hospital, Brooklyn, New York; Columbia University Irving Medical Center, New York, New York; Columbia University, new york, New York; New York Presbyterian Westchester, Mount Kisco, New York; Division of Infectious Diseases, New York–Presbyterian Queens, Flushing, New York; NewYork-Presbyterian Queens, Flushing, New York; Columbia University Irving Medical Center, New York, New York; NEW YORK PRESBYTERIAN, HOBOKEN, New Jersey; NewYork-Presbyterian, Brooklyn, NY; Columbia University Irving Medical Center, New York, New York; NewYork- Presbyterian, New York, New York; Columbia University Irving Medical Center, New York, New York

## Abstract

**Background:**

Hospital-onset bacteremia (HOB) is a quality metric proposed by CDC. HOB caused by methicillin-resistant *Staphylococcal aureus* (MRSA) is reported to Centers for Medicare and Medicaid services through the National Healthcare Safety Network (NHSN), yet few studies have described HOB-MRSA epidemiology. Thus, we report HOB-MRSA epidemiology from 2020-2022 at our multi-hospital health system to inform quality improvement initiatives.

**Figure:**
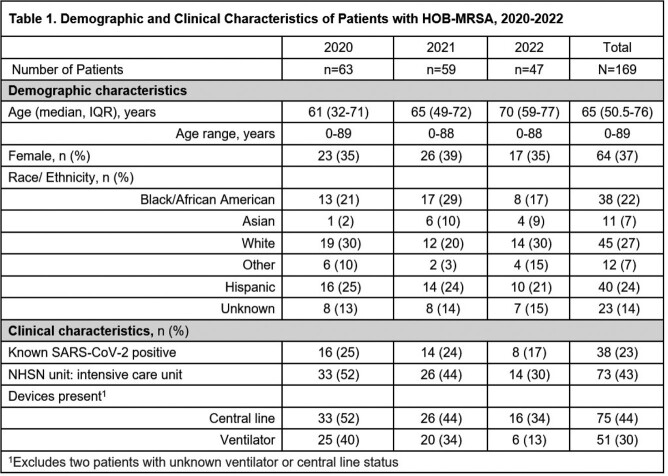

**Figure:**
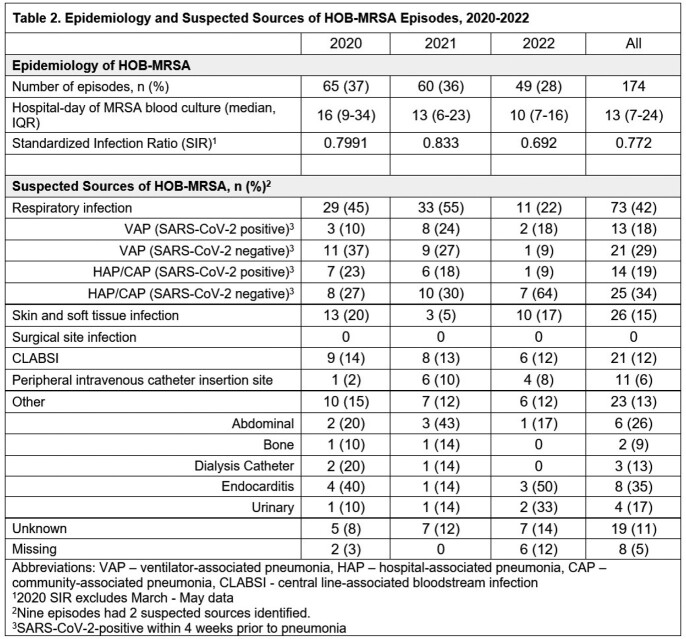

**Methods:**

We used data submitted to the NHSN to prospectively identify HOB-MRSA LabID cases and collected demographic and clinical characteristics from patients’ electronic medical records. Hospital epidemiologists at each of the 9 hospitals adjudicated suspected sources of HOB-MRSA. Annual standardized infection ratios (SIRs) were calculated. Characteristics of HOB-MRSA episodes were analyzed by descriptive statistics. Relevant institutional review boards deemed this study exempt from Human Subjects Research.

**Results:**

From 2020-2022, 174 (0.029%) HOB-MRSA episodes occurred among 573,433 hospitalizations. Table 1 provides the characteristics of patients with HOB-MRSA and Table 2 provides the SIRS and suspected sources of HOB-MRSA. Overall, 43% of patients were in an ICU, 44% had a central line (CL), and 30% were on a ventilator. The SIR was lowest in 2022. Respiratory sources (non-ventilator and ventilator-associated pneumonia (VAP)) were the most common and declined over time (p=0.002). Suspected sources of HOB-MRSA were unknown for 11% of episodes.

**Conclusion:**

We found that episodes of HOB-MRSA were attributed to multiple primary sources of infection. Thus, bundle strategies for VAP and CL-associated bloodstream infections, while crucial, are insufficient to reduce all HOB-MRSA. In addition, prevention strategies for patients with skin and soft tissue infections are needed. Our findings highlight the importance of adherence to all device and procedure-related bundles, hand hygiene, cleaning/disinfection, and MRSA decolonization when appropriate. Elucidating the epidemiology of primary infection sources can further tailor prevention strategies for HOB.

**Disclosures:**

**Sorana Segal-Maurer, MD**, Gilead Sciences, Inc: Advisor/Consultant|Gilead Sciences, Inc: Honoraria|Janssen: Advisor/Consultant|Janssen: Honoraria|Theratechnologies: Advisor/Consultant|Theratechnologies: Honoraria|Viiv: Advisor/Consultant|Viiv: Honoraria **Nishant Prasad, MD**, Gilead Sciences, Inc.: Grant/Research Support **Lisa Saiman, MD MPH**, Merck & Co., Inc,: Grant/Research Support|Merck & Co., Inc,: Member, DSMB|Pfizer, Inc: Member, DSMB

